# Response to Polosa *et al.* Comments on Scheffler *et al.* Cytotoxic Evaluation of E-Liquid Aerosol Using Different Lung Derived Cell Models. *Int. J. Environ. Res. Public Health*, 2015, *12*, 12466-12474

**DOI:** 10.3390/ijerph13010109

**Published:** 2016-01-06

**Authors:** Stefanie Scheffler, Hauke Dieken, Olaf Krischenowski, Michaela Aufderheide

**Affiliations:** Cultex Laboratories GmbH, Feodor-Lynen-Str. 21, 30625 Hannover, Germany; h.dieken@cultex-laboratories.com (H.D.); o.krischenowski@cultex-laboratories.com (O.K.); m.aufderheide@cultex-laboratories.com (M.A.)

Referring to the comments of Polosa and colleagues [1] on our latest *in vitro* e-liquid aerosol testing publication, we would like to give our statements about some points.

In general, we are in agreement with Polosa *et al.* that validated protocols should be the basis for an international test strategy for e-liquids and their aerosols. Due to the upcoming enforcement of the tobacco product directive for e-cigarettes and e-liquids in May 2016, attempts have been made to first establish testing protocols to obtain toxicological data. However, these efforts are limited to chemical data, which might not be sufficient to ensure complete consumer protection in the future. The complexity of the problem cannot be solved by a simple toxicological screening method and should be based on different assays addressing the cytotoxic spectrum of e-liquids and/or their aerosols. In our opinion, it is also necessary to analyze the effects of e-liquid aerosols *in vitro*, since the generated inhalable vapor represents the actual hazardous compounds interacting directly with the epithelium of the respiratory tract. 

In our publication [2], we did not state that the immortalized human bronchial epithelial cell line CL-1548 would be the most suitable candidate for *in vitro* testing of e-liquid aerosols in general. However, due to the fact that the primary impact site of e-liquid aerosol is the respiratory tract, cells from this anatomical region are the most suitable ones. In our opinion, primary cells from healthy human lung tissue would be the most relevant cell model, but due to donor-dependent variations, limited lifespan and limited availability, those cells have their limitations for standard routine testing. Here, immortalized cell lines offer an alternative, because they have unlimited availability and allow testing procedures with comparable cell populations. In our opinion, cytotoxic studies should also not be limited to acute toxicity testing with undifferentiated cells of the respiratory tract, but should also include long-term (chronic) studies on differentiated 3D constructs with all characteristic cell types to address cell-specific cytotoxic effects relevant for the *in vivo* situation. In this context, it is of great importance to have one cell line, which can be used to perform both acute and long-term toxicity studies, in order to obtain a broad spectrum of toxicological data. 

Polosa *et al.* [1] mentioned that different fully characterized human bronchial epithelial cell lines are available from ATCC like BEAS-2B and 16HBE14o- cells. Here, it has to be mentioned that the virus-transformed BEAS-2B cells do not exhibit a differentiation comparable to that of their parent cells, lack tight junctions [3] and become malignant after several passages [4]. The also named 16HBE14o- cells, also virus-transformed, are not able to differentiate into a pseudostratified airway epithelium under submersed as well as air–liquid interface conditions [5]. In our studies, we integrated a cell line which has been immortalized at SIRION BIOTECH GmbH (Germany) using lentiviral constructs containing cyclin-dependent kinase (CDK4) and human telomerase reverse transcriptase (hTERT), which shows comparable morphological characteristics of the donor cells (ciliated and mucus-producing as well as progenitor cells). 

We compared the cellular effects (viability and the production of reactive oxygen species) after e-liquid aerosol and mainstream smoke exposure on freshly isolated primary bronchial epithelial cells, the immortalized cell line CL-1548 and the alveolar cell line A549 [2]. Our experiments demonstrated that A549 cells exhibit a significantly lower susceptibility to cellular damage than the primary cells and also the reaction pattern between the different exposure groups is not comparable with them, whereas it is comparable for freshly isolated bronchial epithelial and immortalized CL-1548 cells. These results clarify that A549 cells have a different response characteristic to that of the primary cells. Based on the assumption that primary cells should be set as the “gold standard” for cytotoxic evaluation, a cell line used for routine tests should give results as close as possible to this standard. 

Regarding Polosa *et al.*’s [1] notes about the experimental design, we would like to explain our study approach. Since there are no standard protocols for e-cigarette testing as yet, we decided to work according to ISO 3308 and compared the toxicity of cigarette mainstream smoke to e-liquid vapor. In order to be able to compare the results of both exposures (e-liquid aerosol/mainstream smoke), we used the same smoking protocol for e-cigarettes as for combustible cigarettes, generated dose-response curves dependent on the number of puffs during the exposure and chose for our experiments a “dose” of 200 puffs for e-liquid aerosols. However, a decrease in cell viability was seen already after the exposure to 50 puffs. In the case of mainstream cigarette smoke, 60 puffs induced a strong cytotoxicity (about 80%) and a further increase in the number of puffs resulted in complete cell death. Accordingly, only the consideration of 60 puffs or less allows a theoretical comparison of the results, presented on a puff-to-puff comparison. Such an adjustment of the results was possible due to the linear dose-response relationships in both cases (cigarette and e-cigarette exposure).

In our case, we did not work according to the standard protocol (ISO 10993-5) for testing substance extracts *in vitro*, because testing extracts under submersed culture conditions does not reflect the situation after vaping/smoking *in vivo*. In the lung, the cells are not covered with a liquid layer as found during submersed cultivation, but are exposed directly to the surrounding atmosphere. Furthermore, water-soluble and volatile vapor components cannot be trapped in the extracts and are therefore not analyzed during the testing. 

In summary, we are convinced that direct exposure studies with normal human bronchial epithelial cells or relevant immortalized cell lines in an undifferentiated as well as differentiated stage will contribute to the evaluation of the cytotoxic potency of e-liquid vapor. Such investigations should be included in a validated research protocol accepted by international regulatory authorities.
